# Overexpression of *KvCHX* Enhances Salt Tolerance in *Arabidopsis thaliana* Seedlings

**DOI:** 10.3390/cimb45120605

**Published:** 2023-12-01

**Authors:** Yuqi Guo, Chengrong Zhu, Zengyuan Tian

**Affiliations:** 1 School of Life Sciences, Zhengzhou University, Zhengzhou 450001, China; guo_yuqi2021@126.com (Y.G.); 18856300358@163.com (C.Z.); 2 School of Agricultural Sciences, Zhengzhou University, Zhengzhou 450001, China

**Keywords:** *Kosteletzkya virginica*, *KvCHX*, transgenic arabidopsis, salt stress, K^+^ transport

## Abstract

The CHX (cation/H^+^ exchanger) family plays an important role in the transmembrane transport of cation/H^+^ in plants. The aim of this study was to identify and functionally analyze the *KvCHX* gene in the halophyte *Kosteletzkya virginica* to investigate its role in regulating the K^+^/Na^+^ ratio under salinity tolerance. Based on a partial gene sequence of EST from *K. virginica*, the full-length DNA sequence of the *KvCHX* gene was obtained using genome walking technology. Structural analysis and phylogenetic relationship analysis showed that the *KvCHX* gene was closely related to the *AtCHX17* gene. The *KvCHX* overexpression vector was successfully constructed and transformed into Arabidopsis via floral dipping. Arabidopsis seedlings overexpressing *KvCHX* showed an enhanced tolerance to salt stress compared with wild-type plants. Transgenic Arabidopsis seedlings grew better under K^+^ deficiency than WT. The results showed that *KvCHX* could promote the uptake of K^+^, increase the ratio of K^+^/Na^+^, and promote the growth of plants under K^+^ deficiency and treatment with NaCl solution. *KvCHX* is involved in K^+^ transport and improves plant salt tolerance by coordinating K^+^ acquisition and homeostasis.

## 1. Introduction

Plants are often affected by different environments, such as high salt levels, drought, and extreme temperatures [1]. Salt stress is the main abiotic factor affecting plant growth and development. Currently, at least 800 million hectares of land in the world are affected by salinization, which is more than 6% of the world’s total land area [2]. Generally, soils with a soil solution electrical conductivity (EC) of 4 dS/m (deciSiemens per meter) or greater are referred to as saline soils [3]. Plants are classified into glycophytes and halophytes based on their ability to grow in highly saline soils. Halophytes have a high salinity tolerance and can survive and reproduce in environments with salt concentrations around or above 200 mm NaCl [4]. In contrast, at high salt concentrations [5], glycophytes are subjected to ionic, osmotic, and secondary stresses.

The major effect of salt stress is the accumulation of Na^+^ and Cl^−^ ions in plant tissues exposed to saline soils [6]. The massive uptake of Na^+^ and Cl^−^ results in a severe ionic imbalance. High Na^+^ concentrations inhibit the uptake of K^+^ ions, which are important elements for growth and development, and low K^+^ concentrations lead to decreased productivity and even death [7]. To ensure survival in this adverse environment, halophytes have evolved their own mechanisms for salt tolerance. Some halophytes reduce harmful ion concentrations by removing harmful ions from roots and shoots through salt glands, and some halophytes are able to tolerate high concentrations of salt, while others adopt measures such as ion compartments, osmoregulation, and the synthesis of compatible solutes [3,4]. Therefore, the salinity tolerance of halophytes depends on the controlled uptake and fractionation of Na^+^, K^+^, and Cl^−^ and the synthesis of organic solutes [4]. Among them, K^+^ is the most abundant cation in plant cells, accounting for about 10% of the dry weight of plants [8]. Potassium ions mediate many physiological reactions in plants, such as stomatal closure and opening, leaf movement, and plant tropism driven by swelling pressure caused by K^+^ [9,10,11]. High concentrations of Na^+^ are toxic to plants and have a negative effect on plant growth, but Na^+^ is greater than K^+^ in saline soils. Therefore, faced with competition from Na^+^ [12], halophytes must choose to absorb K^+^.

To regulate K^+^ uptake and balance, plants have evolved transport systems containing a large number of transporters, such as HAK/KUP/KT (K^+^/H^+^ transporter), HKT/TRK (K^+^/H^+^ or K^+^/Na^+^ transporter), CPA (cation/H^+^ anti-transporter), and Shaker channels. At present, some *CHX* genes in Arabidopsis have been revealed to have their functions through heterologous expression. They may play an important role in regulating membrane cation and pH homeostasis [13]. *AtCHX20* has been confirmed as a K^+^ transporter for guard cells [14]. Similarly, in the latest research, the *GmSALT3* gene in soybeans has been confirmed to be an endometrial localization protein with transport ability and plays an important role in salt tolerance [15]. *Ksteletzkya virginica* is a dicotyledon halophyte (http://plants.usda.gov, accessed on 26 October 2023) native to the southeastern United States, which often grows in coastal soils containing 0.3–2.5% sodium salts (mainly NaCl) [16]. As a seaside mallow, *K. virginica* is a perennial dicotylote and halophyte native to brackish portions of coastal tidal marshes in the mid-Atlantic and southeastern United States and is considered an obligate wetland species. It extends north to Long Island, New York, south to Florida, and along the eastern Texas coastal plain [17]. Blanchard synonymized *K. Pentacarpos* (found in Eurasia) with *K. virginica*. With a lifespan of about 11 years and a high seed protein (32%) and oil (22%) content, *K. virginica* is attractive not only for animal feed and biomass production in coastal areas unsuitable for conventional agriculture [18], but also for biodiesel and ethanol production [19]. In 1992, *K. virginica* was introduced into Jiangsu Province, China. *K. virginica* can be grown in saline or arid lands and can be irrigated with salt or sea water. The salt physiological properties of *K. virginica* were investigated and the results showed its high salt tolerance [20]. However, the specific mechanism of salinity tolerance in *K. virginica* remains elusive. In a previous report, we found the expression of *K. virginica CHX* (*KvCHX*) was up-regulated in roots under salt stress and obtained the EST of the *KvCHX* gene [21]. Therefore, we propose a hypothesis that *KvCHX* may be involved in the salinity tolerance of *K. Virginia*. Previous studies have shown that comparing the AtCHX protein with fungal and bacterial CPA2 proteins reveals that the CHX protein has K^+^, Na^+^, and H^+^ transport functions [22]. Moreover, after phylogenetic analysis of the plant CHX gene family, it was found that it has important genetic relationships with many ion transport genes [23]. Here, we verified the function of the *CHX* gene of *K. virgineca*, which is associated with its ability to maintain Na^+^ and K^+^ homeostasis in transgenic Arabidopsis seedlings.

In this study, a DNA sequence of *KvCHX* was obtained based on a *KvCHX* known fragment [21] using genome walking technology. Protein structure analysis showed that the 2400 bp CDS of *KvCHX* encodes a polypeptide containing 799 amino acids. The predicted KvCHX protein consists of an amino-terminal domain with 12 transmembrane spans (430 residues) and a hydrophilic domain at the carboxyl end, which is proposed to have regulatory roles. Subcellular localization analysis showed that KvCHX was localized to the cytoplasmic membrane. Phylogenetic relationship analysis implied that KvCHX was very similar to AtCHX17; both of them shared higher identity in amino-terminal hydrophobic domains than other members of the *AtCHX* gene family, especially TM5 and TM11 (91.30% and 90.00% identity, respectively). To investigate the role of *KvCHX* in K^+^ transport and salinity tolerance, an overexpression vector 2×35S-KvCHX-Nos was constructed and transformed into *Arabidopsis thaliana* ecotype Columbia (WT) using floral dipping. The T_3_ transgenic lines of Arabidopsis seedlings could promote the absorption of K^+^, increase the K^+^/Na^+^ ratio, and contribute to the growth of plants under K^+^ deficient conditions and treatment with NaCl solution. In summary, the results indicated that *KvCHX* enhances salt tolerance in transgenic Arabidopsis by regulating the K^+^/Na^+^ ratio under salt stress.

## 2. Materials and Methods

### 2.1. Plant Materials and Stress Treatments

*A. thaliana* ecotype Columbia (WT) was used in this study. Seeds of *K. virginica* were soaked overnight in fresh water and then sown in pots filled with vermiculite in a greenhouse at 26/20 °C with day/night of 15/9 h.

WT and T_3_ transgenic Arabidopsis seeds were sterilized with sodium hypochlorite and placed in MS medium for 2 weeks. Seedlings (5 plants per treatment) were grown under hydroponic conditions in a greenhouse. For greenhouse cultivation, seedlings used in the transgenic experiment were transplanted into pots filled with vermiculite and soil (3:1) and watered with Hoagland solution. For hydroponic cultivation, the seedlings were transferred to nutrient solution containing 1 mM Ca(NO_3_)_2_, 1 mM KH_2_PO_4_, 1 mM KNO_3_, 1 mM MgSO_4_, 50 μM Na-Fe-EDTA, 50 μM H_3_BO_3_, 0.05 μM CoCl, 0.05 μM CuSO_4_, 15 μM ZnSO_4_, 50 μM MnSO_4_, and 3 μM Na_2_MoO_4_ [24]. The environmental parameters in the growth chamber were as follows: light/dark cycle of 8/16 h, light intensity of 300 μmol s^−1^m^−2^par, temperature of 24 °C/22 °C, humidity of 70%. The nutrient solution was renewed once a week during the first part of the culture and twice a week during the experiment.

In the K^+^ deficient treatment, plants (five plants per treatment) were selected at the rosette stage at the beginning of each experiment [25]. Plant roots were rinsed in 0.2 mM CaSO_4_ for 5 min and then transferred to 1/2 K^+^ nutrient solution. In 1/2 K^+^ nutrient solution, KH_2_PO_4_ was replaced by 1 mM NaH_2_PO_4_. In the salt treatment, the basal nutrient solution was supplemented with 0 mM, 100 mM, 150 mM, and 200 mM NaCl, respectively. 

### 2.2. Isolation and Sequence Analysis of KvCHX, Protein Structure, Phylogenetic Tree, and Multiple Alignment

Genomic DNA of *K. Virginica* was extracted from 10-day-old seedlings using the NuClean PlantGen DNA Kit (Biotech, Hefei, China) according to the manufacturer’s instructions and examined via electrophoresis on 1.5% agarose gels.

To obtain the full-length DNA sequence of *KvCHX*, the 5′ sequence was extended three times, and the 3′ sequence once, via TAIL PCR, using the genome Walking toolkit (Takara D316) based on the known fragment of the *KvCHX* gene (FK816439). Gene-specific primers (GSPs) were designed each time based on the obtained sequences using genomic DNA as a template (Table 1). For each genome walking, three rounds of TAIL-PCR (Thermal Asymmetric Interlaced PCR) were performed using the GSPs and degenerate primers for the 5′-end flanking sequence of the known segment of the *CHX* gene.

This technique is well suited to cloning the flanking sequences of genes based on the partial known sequences of genes, especially when the species under study has not yet been fully sequenced. A total of three gene-specific primers (GSPs) close to the flanking sequences at the 5′ end of known fragments were designed and synthesized sequentially. The primer GSP1 is upstream of GSP2, and the primer GSP2 is upstream of GSP3. TAIL-PCR was performed with four arbitrary degenerate primers combined with specific primers (GSP3, GSP2, and GSP1). For each chromosome walk, TAIL-PCR, including three rounds of PCR, was performed using three specific primers designed according to known sequences, which were designated as GSP1-5′-1~GSP1-5′-3, GSP2-5′-1~GSP2-5′-3, and GSP3-5′-1~GSP3-5′-3, respectively, for 5′-end flanking cloning every time, and GSP1-3′-1~GSP1-3′-3 for 3′-end flanking cloning one time only. Firstly, four arbitrary degenerate primers (AP primers) in combination with the specific primer GSP3 were used for first TAIL-PCR. Three rounds of TAIL-PCR were carried out on a Biorad Thermal Cycler. The products from the first round of TAIL-PCR were used as templates for the secondary PCR, and the products from the second round of TAIL-PCR were used as templates for the third round of PCR, and were then isolated from agarose and cloned into the T vector for nucleotide sequencing. Secondly, the specific primer GSP2 was designed based on the above sequencing results, and the second chromosome walk was further performed on the basis of the first. A final chromosome walk was then performed using the GSP1 primer dependent on the results of the second sequencing to obtain the 5′-end flanking sequence. Each product from each of the third TAIL-PCR rounds was separated on an agarose gel and sequenced. The resulting target sequence was determined vis analyzing the overlapping amplified fragments of the three 5′-end flanking discrete PCR products. Finally, the 3′-end flanking sequences of the known segments of the *CHX* gene were isolated using TAIL-PCR with GSP1-3′. The volume of a single PCR was 50 μL, containing 2 μL of genomic DNA, 5 μL of 10 × LA PCR BufferII (Mg^+^ plus), 8 μL of dNTP mixture (2.5 mM each), 0.5 μL TakaRa LA Taq (5 U/μL), 1uL of AP primer (100 pmol/μL), 1 μL of GSP primer (10 pmol/μL), and 32.5 μL of ddH_2_O. Table 2 summarizes the thermal cycling conditions. PCR products were detected via 2% agarose gel electrophoresis and purified (AxyPrepTM DNA Gel Extraction kit) and cloned into PMD18-T vector (TaKaRa, Beijing, China) for sequencing. The full-length *KvCHX* gene sequence was obtained.

Gene structure prediction was analyzed using SoftBerry FGENESH (http://www.softberry.com/berry.phtml, accessed on 26 October 2023). Protein prediction was performed using the ExPASy server (http://web.expasy.org/protparam/, accessed on 26 October 2023). Signal sequence analysis and nuclear localization signal analysis were performed using the SignalP 4.1 server and NLS prediction, respectively. ProtComp 9.0 was used for subcellular localization analysis, and TMHMM Server 2.0 for investigating the transmembrane domain. SWISS-MODEL was used for protein tertiary structure prediction. The multiple sequence alignment was performed between *AtCHX*s and *KvCHX* using ClastalX (1.81) for homology analysis, and the phylogenetic tree was constructed using MEGA4.1 to compare the evolutionary relationships of *KvCHX* with *AtCHX*s.

### 2.3. Vector Construction and Transformation

The *KvCHX* gene was amplified via PCR from *K. virginica* genome DNA using the primers of *CHX*-F (5′-CACCGGCAGGGGAAGTGAAATC-3′) and *CHX*-R (5′-TGTTGTTTCTA CATATCCTTCG-3′). The transformation vector was constructed using Gateway^®^ Technology with the ClonaseTM Kit (Invitrogen, Carlsbad, CA, USA) according to the manufacturer’s protocol (www.invitrogen.com/gateway, accessed on 26 October 2023) [26]. The recombinant plasmid P2×35S::GFP-KvCHX was introduced into Agrobacterium tumefaciens strains GV3101 [27] using the liquid nitrogen freeze–thaw method [28]. Arabidopsis was transformed with A. tumefaciens transformation containing the P2×35S::GFP-CHX plasmid using floral dipping technique [29]. Transgenic Arabidopsis was grown in a greenhouse at 24/22 °C, 70% relative humidity, and a 16/8 h light/dark cycle. Transformed Arabidopsis seeds were harvested from transformed plants (T_0_) and grown on MS medium containing 50 mg L^−1^ hygromycin. Hygromycin-resistant lines were selected for PCR identification. Homozygous T_3_ progeny from the T_2_ population were selected and further verified via Western blot and salt tolerance analyses [30].

### 2.4. Confirmation of Transgenic Arabidopsis Plant

Genomic DNA was extracted from 6-week-old seedling leaves of T_3_ transgenic plants and wild-type control via CTAB. PCR was performed using primers GFP-FP (5′-ATGAGTAAAGGAGAAGAACTTTTCACTG GA-3′) and CHX-R2 (5′-CATTTGTGTTTGTATCGG CTGC-3), and the amplification was performed using the program: 94 °C for 2 min, followed by 35 cycles of program (94 °C for 30 s, 60 °C for 30 s, 72 °C for 2 min), and terminated with an extension at 72 °C for 5 min. The PCR products were detected via electrophoresis on 1.5% agarose gel.

The PCR amplification was carried out using genomic DNA extracted from 6-week-old seedling leaves of T_3_ transgenic plants and wild-type control via CTAB. The primers used for PCR were GFP-FP (5′-ATGAGTAAAGGAGAAGAACTTTTCACTGGA-3′) and CHX-R2 (5′-CATTTGTGTTTGTATCGG CTGC-3′). The PCR program consisted of an initial denaturation at 94 °C for 2 min, followed by 35 cycles of denaturation at 94 °C for 30 s, annealing at 60 °C for 30 s, and extension at 72 °C for 2 min, and a final extension at 72 °C for 5 min. The PCR products were then analyzed via electrophoresis on a 1.5% agarose gel.

### 2.5. Western Blot Analysis of Transgenic Arabidopsis Plant

WT and transgenic Arabidopsis plants were grown under hydroponic conditions in basal nutrient solution. After 10 days of cultivation, the seedlings were transferred to nutrient solution for 10 days. Total protein was extracted from WT and transgenic Arabidopsis seedlings with a buffer consisting of 50 mM Tris/HCl (pH 8.0), 150 mM NaCl, 1 mM EDTA, and 0.2% (*w/v*) Triton X-100, 4% β-mercaptoethanol, 1 mM dithiothreitol (DTT), and 1% (*v/v*) protease inhibitor cocktail (BBI Life Science, Shanghai, China) and then used for protein quantification with BCA protein quantitative Kit (Boster, Pleasanton, CA, USA). The protein samples (200 μg amounts) were electrophoresed in 8% SDS-PAGE, and the gels were transferred to nitrocellulose membranes. The membranes were blocked with TBST buffer (10 mM Tris/HCl (pH 7.5), 150 mM NaCl, and 0.05% Tween-20) supplemented with 5% non-fat milk for 2 h and incubated with primary antibodies (Anti-GFP antibody, Abcam (Cambridge, UK), diluted at 1:200) in TBST buffer with 5% BSA overnight at 4 °C. Afterwards, the membranes were washed three times (10 min each) with TBST buffer and incubated with the secondary antibodies (Goat Anti-Mouse IgG H&L (HRP), Abcam, dilution at 1:1000) for 2 h. After being washed three times with TBST buffer, the membranes were incubated with a chromogenic agent Enhanced HRP-DAB Chromogenic Substrate Kit (Boster).

### 2.6. Subcellular Localization Assay

Fluorescence of KvCHX-GFP fusion protein in transgenic plants was observed using confocal laser-scanning microscopes. The roots and leaves of 4-week-old transgenic plants were selected, and the epidermis was removed for observation.

### 2.7. Salt Stress Treatments Involving the Transgenic Arabidopsis Plant

Seeds of transgenic and WT plants were sterilized with sodium hypochlorite and planted on MS medium for 2 weeks. Then, the seedlings were transferred into a greenhouse and fixed on polyvinyl chloride (PVC) plates folating on 1/2 Hogland nutrient solution supplemented with 0 mM, 100 mM, 150 mM, and 200 mM NaCl, respectively, for 7 days. The salt treatment nutrient solution was changed every 3 days, and after 7 days of treatment, the growth conditions of seedling, such as root length and leaf number, and physiological indexes were measured. The proline content of WT and transgenic plants was measured as described by [31], and the malondialdehyde (MDA) content was determined according to the method of Song [32].

### 2.8. K^+^ Deficient Treatments of Transgenic Arabidopsis Plant

Seeds of transgenic Arabidopsis plants and WT were collected and sterilized with sodium hypochlorite, then planted in MS medium for germinating and cultivating for 10 days. Then, WT and transgenic Arabidopsis plants were transferred into Hogland nutrient solution with 1/2 K^+^ (KNO_3_) concentration for 20 days. The leaf number, branch number, fresh weight, and dry weight were determined.

### 2.9. Determination of Potassium and Sodium Ion Content

The second fully expanded rosette leaves and the mature zone of the roots (without the root tip) were collected and washed in 0.2 mM CaSO_4_ for 5 min. To quantify the potassium and sodium ion content, tissues harvested from five plants per treatment were oven-dried at 100 °C for 2 h and at 85 °C for 18 h. Subsequently, the plant material was digested with HNO_3_ at 80 °C and dissolved with ddH_2_O for ion extraction. The concentrations of cation, sodium, and potassium ions in tissue samples were determined using a Thermo fisher ICP spectrometer. All measurements were performed in triplicate.

## 3. Results

### 3.1. Isolation and Sequence Analysis of KvCHX

**Figure 1:**
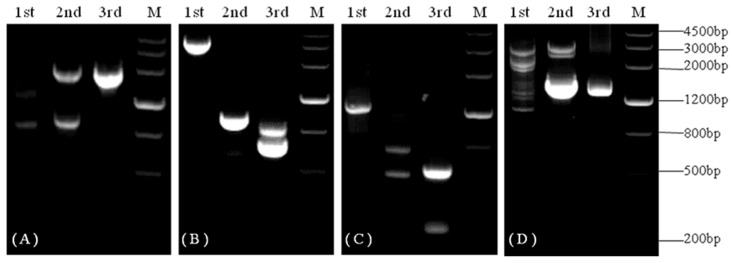
PCR products during the genome walking of *KvCHX*. (**A**–**D**) represent the genome walking products of the 3′-end sequence and the third, the second, and the first genome walking for the 5′-end sequence, respectively.

BLASTX analysis of the 496 bp known fragment of *KvCHX* (FK816439) revealed high similarity between the *KvCHX* fragment and *CHX*s described in other species, such as cocoa, cherry, grape, tomato, corn, and Arabidopsis. To obtain the full-length *KvCHX* sequence, three rounds of TAIL-PCR were performed with template genomic DNA and the primers GSP1-5′-1~GSP1-5′-3, GSP1-3′-1~GSP1-3′-3 for 5′ flanking extension firstly and 3′ flanking extension, respectively. The PCR products were examined via electrophoresis on 2% agarose gel, yielding two fragments of approximately 1800 bp (Figure 1A) and 1400 bp (Figure 1D). The final round of TAIL-PCR products were cloned into the pMD18-T vector and sequenced. Sequence analysis (BioXM 2.6) showed that the overlapping regions of these fragments were the same as those of known *KvCHX* fragments. These three fragments include the known *KvCHX* fragment and the two sequenced fragments above, and we obtained a fragment of about 3440 bp via assembly. BLASTX analysis showed that the amino acid sequence of this fragment was significantly similar to that of the CHX proteins of cacao, prunus persica, and grape.

Via the second genome walking for the 5′ flanking-sequence, we obtained two fragments, of approximately 800 bp and 600 bp, respectively. Sequencing analysis showed that the 670 bp fragment was a specific product (Figure 1B, 3rd). The last genome walking for the 5′ flanking-sequence allowed us to obtain a fragment of approximately 611bp (Figure 1C, 3rd). Finally, we obtained a 4333 bp fragment by assembling these fragments (the 496 bp, 3440 bp, 670 bp, and 611 bp fragments). Sequence analysis suggested that it encodes a complete protein.

To further obtain the assembled 3440 bp upstream of the 5′ flanking-sequence, genome walking was performed twice based on the obtained 5′ flanking sequence. The second genome walking for the 5′ flanking sequence yielded two fragments, of approximately 800 bp and 600 bp, respectively. Sequencing analysis showed that the 670bp fragment was a specific product (Figure 1B, 3rd). The last genome walking of the 5′ flanking sequence allowed us to acquire a fragment of approximately 611 bp (Figure 1C, 3rd). Finally, we assembled these fragments (496 bp, 3440 bp, 670 bp, and 611 bp) together to obtain a fragment of 4333 bp. Sequence analysis showed that it encodes a complete protein.

### 3.2. Analysis of KvCHX Gene Structure

**Figure 2:**
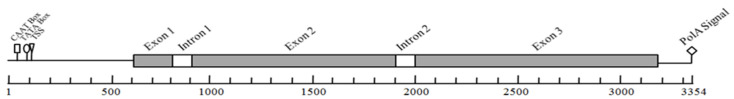
Structural sketch of the *KvCHX* gene.

The full-length open reading frame (ORF) of *KvCHX* was analyzed using SoftBerry FGENESH and BLASTX (Figure 2), and the results showed that the *KvCHX* gene had an ORF of 2562 bp (from 627 bp to 3189 bp). There were three exons (627 bp–827 bp, 913 bp–1914 bp, and 1993 bp–3189 bp, respectively) and two introns (828 bp–912 bp and 1915 bp–1992 bp, respectively). The CAAT Box and TATA Box were located at 50 bp and 100 bp, respectively. The TSS was located at 125 bp, and the Poly (A) tail of the mRNA was located at 3354 bp.

### 3.3. Structure and Function Analysis of KvCHX Protein

**Figure 3:**
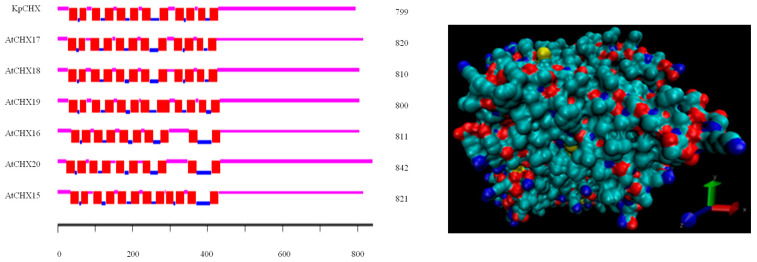
Transmembrane spans of the predicted amino acid sequences of KvCHX and AtCHX15~AtCHX20 (left) and tertiary structure prediction of the KvCHX protein from different perspectives using SWISS-MODEL. The protein visualization model is a single atom drawing method, where O is marked in red, N is marked in blue, C is marked in cyan, and S is marked in yellow (right).

The physical and chemical properties of the *KvCHX* protein were analyzed using the ProtParam program. The results showed that *KvCHX* encoded a polypeptide of 799 amino acids with a molecular weight of 86.6 kDa and a theoretical pI of 8.86. Its molecular formula was C_3934_H_6355_N_1023_O_1097_S_33_. The *KvCHX* protein was predicted to be a hydrophobic basic lipid-soluble protein. Signal sequence analysis and nuclear localization signal analysis showed that the *KvCHX* protein had no signal peptide and nuclear localization signal. Transmembrane domain analysis revealed that *KvCHX* has 12 putative transmembrane domains in a highly hydrophobic N-terminal region (Figure 3). Subcellular localization analysis showed that the protein was located on the plasma membrane.

### 3.4. Phylogenetic Analysis and Multiple Alignment of CHXs

**Figure 4:**
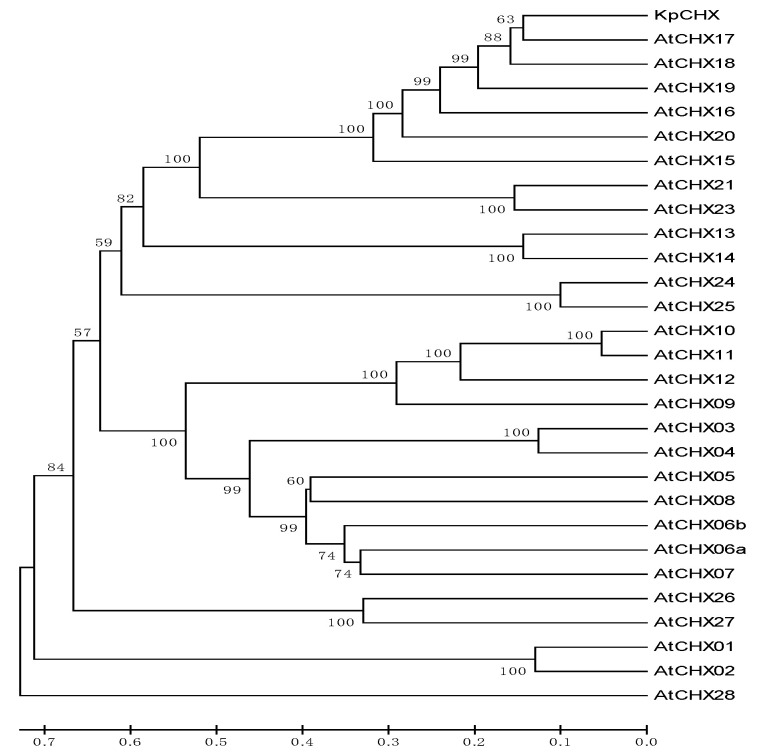
Phylogenetic relationship among *KvCHX* and 28 members of the *Arabidopsis thaliana* CHX family. *AtCHX1*, AY926465.1; *AtCHX2*, AEE36238.1; *AtCHX03*, AY926466.1; *AtCHX04*, DQ499020.1; *AtCHX05*, NP_172294.2; *AtCHX06a*, AEE28253.1; *AtCHX06b*, AEE28251.1; *AtCHX07*, AEC08087.1; *AtCHX08*, AY926468.1; *AtCHX09*; AY926469.1; *AtCHX10*, AEE77969.1; *AtCHX11*, AEE77968.1; *AtCHX12*, AEE77967.1; *AtCHX13*, EFH573351.1; *AtCHX14*, EFH65886.1; *AtCHX15*, AEC06246.1; *AtCHX16*, AEE34204.1; *AtCHX17*, EFH43951.1; *AtCHX18*, EFH44842.1; *AtCHX19*, ANM63400.1; *AtCHX20*, ANM63347.1; *AtCHX21*, ANM62431.1; *AtCHX23*, AEE27860.1; *AtCHX24*, EFH44758.1; *AtCHX25*, EFH42526.1; *AtCHX26*, AED90376.1; *AtCHX27*, AD90377.2; *AtCHX28*, AEE7888.1.

To compare the evolutionary relationships, the putative *KvCHX* and *AtCHX* family were used to construct the phylogenetic tree using MEGA-X with the neighbor-joining (NJ) method and 1000 bootstrap replicates. The phylogenetic tree showed that a total of 28 members of the Arabidopsis *CHX* family could be divided into five subclades [21], among which *AtCHX15-AtCHX20*, *AtCHX21*, and *AtCHX23* belonged to the fourth class, and the *KvCHX* gene was closely related to the *AtCHX17* gene (Figure 4).

**Figure 5:**
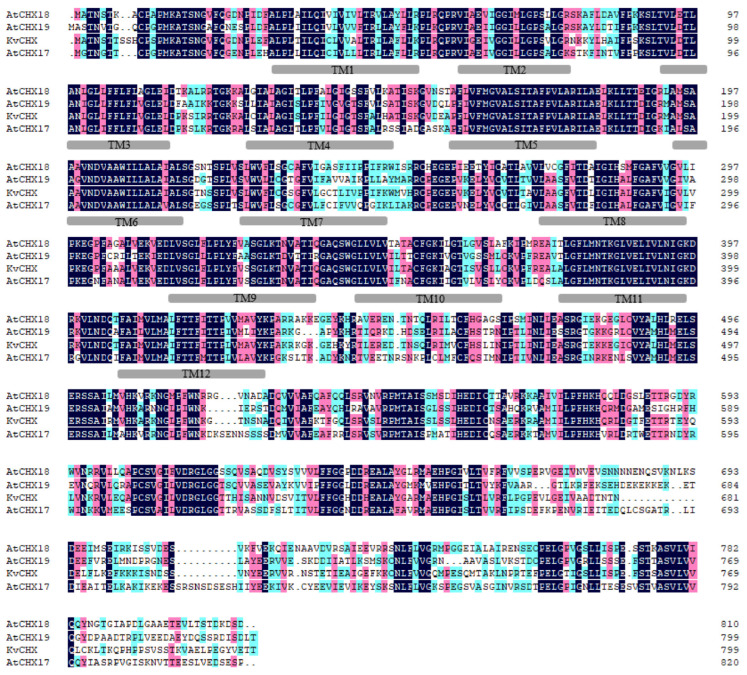
Multiple amino acid sequence alignments of *KvCHX* and *AtCHX17–19*. Identical amino acids are shaded in black. Similar amino acid residues are shaded in pink and light blue.

To further reveal the structure of the *KvCHX* sequence, the software ClastalX (1.81) was used to align the *KvHXK* sequence and *AtCHX17–19* members as the reference to find the conserved domain, so as to determine the similarity between the AtCHX protein sequence and *KvCHX*. As a result, similar to *AtCHX* family members *AtCHX*(17~19), the hydrophobic domain of *KvCHX* contains 12 conserved transmembrane domains, including 430 N-terminal amino acid residues, and a C-terminal hydrophilic domain located outside the plasma membrane, which was predicted to have a regulatory role (Figure 5). Multiple sequence analysis also showed that *KvCHX* and *AtCHX 17–19* had a high identity in the N-terminal hydrophobic transmembrane domain, and the identity level was 80.00%, 79.31% and 77.01%, respectively. In addition, among the 12 conserved transmembrane domains, 21 out of 23 amino acid residues in the TM5 domain, and 18 out of 23 amino acid residues in the TM11 domain, were completely identical, with 91.30% and 90% identity levels to homologous sequences, respectively. This indicated that TM5 and TM11 may play a greater direct role in restoring cation/H^+^ transport.

### 3.5. Expression and Subcellular Localization of Transgenic A. Thaliana

**Figure 6:**
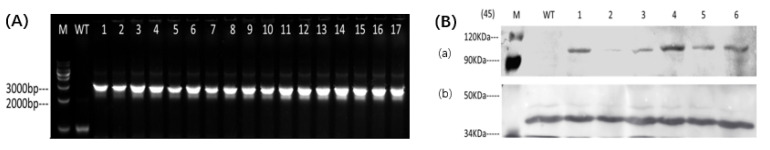

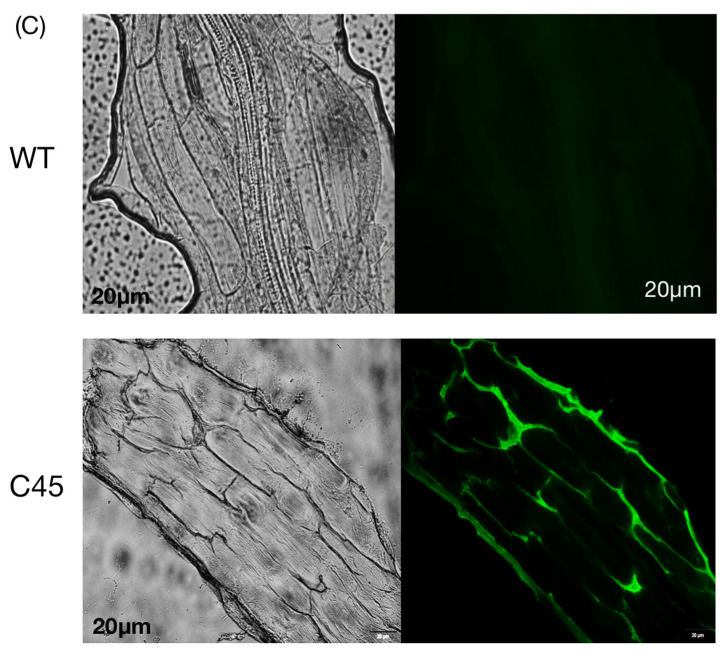
(**A**) PCR product of WT and transgenic Arabidopsis lines. M, 1 Kb Ladder; WT, wild type; 1–17, the transgenic lines. (**B**) Western blot analysis of transgenic Arabidopsis. M, protein marker; WT, wild type; 1–6, C45 transgenic lines. (a) KpCHX-GFP protein; (b) GAPDH as reference protein. (**C**) The GFP fluorescence observation of the root in WT and transgenic plant seedlings (C45) (GFP: at 488 nm excitation and emitted between 515 and 565 nm).

To investigate the function of the *KvHXK* gene under salt stress, the pMDC45 vector containing GFP-KvCHX was transformed into A. tumefaciens strain GV3101, and then successfully transferred into Arabidopsis plants via the floral dipping technique. To check the integration of the *KvCHX* gene in different transgenic lines, PCR analysis used to amplify the integrated fragment was performed using genomic DNA from leaves from WT and T_3_ transgenic seedlings. The bands of the integrated fragment of 2978 bp predicted were displayed (Figure 6A). Our result showed that the *KvCHX* gene from 17 seedlings had been integrated into the genome of *A. thaliana*.

The GFP-KvCHX fusion protein in transgenic Arabidopsis seedlings was analyzed for the expression of the *KvCHX* gene. The KpCHX protein had a molecular weight of 86.6 KDa, and together with the GFP’s molecular weight of 27 KDa, it was expected to form a 113 KDa fusion protein. Western blot analysis showed that there was one obvious protein band between 90 KDa and 120 KDa. The band close to 120 KDa was predicted as the target band (Figure 6B). Therefore, the KvCHX protein was successfully detected in transgenic plants under salt stress and 1/2 K^+^ deficiency treatments.

To investigate the subcellular localization of *KvCHX*, the young root tip and leaf of transgenic seedlings (designated as C45) and WT were selected to observe the fluorescence. A green fluorescence signal was present in the plasma membrane (Figure 6C). The result showed that the KvCHX protein exclusively localized to the plasma membrane. This result was consistent with the *KvCHX* structure using the in silico approaches mentioned above.

### 3.6. Overexpression of KvCHX Enhances Tolerance to Salt Stress in Transgenic Arabidopsis

**Figure 7:**
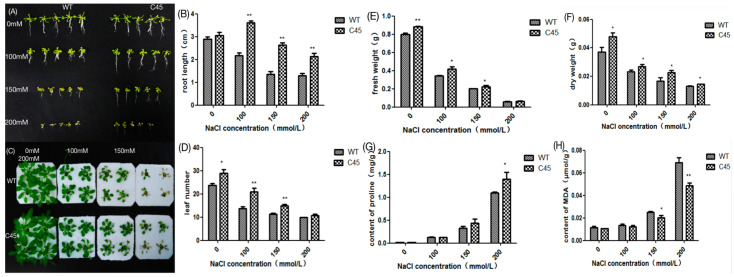
Effects of salt stress on WT and transgenic Arabidopsis lines. (**A**) Seedings of WT and C45 transgenic Arabidopsis in MS medium supplemented with NaCl. (**B**) Root length of (**A**). (**C**) Seedlings of transgenic Arabidopsis (C45) and WT were planted in basic nutrient solution to rosette growth stage, and then transferred into nutrient solution supplemented with 0 mM, 100 mM, 150 mM, and 200 mM NaCl for 7 days. (**D**–**H**) The leaf numbers, fresh and dry weight, MDA content, and proline content of (**C**), respectively. (Values are mean ± SD for three independent replicates, *n* = 5.) *, *p* < 0.05; **, *p* < 0.01 in two-tailed Student’s *t*-tests.

Through there was no significant difference in germination rate between WT and transgenic plants (C45) cultured in MS medium, there was a significant difference in phenotype. Under salt stress treatment, root elongation was severely retarded (Figure 7A,B), and leaves gradually lost greenness (Figure 7C,D). Compared with WT, transgenic plants showed particularly vigorous root development, and leaves remained green under a 150 mM NaCl concentration of salt stress. With increasing NaCl concentration, the root length, fresh weight, and dry weight of transgenic plants and WT all decreased to varying degrees (Figure 7B,E,F). It was noteworthy that the root length and fresh and dry weight of transgenic plants, however, were significantly higher than those of WT plants at the same NaCl concentration. In the same concentration of NaCl solution, transgenic plants had significantly more leaves than WT plants (Figure 7C,D). In addition, prolines are an important indicator that represents the extent to which a plant is tolerant to salt stress; our results suggested that the proline content of transgenic plants is higher than that of WT under salt stress, especially in 200 mM NaCl. As an important indicator of salt tolerance, transgenic plants had a higher proline content than wild-type plants under salt stress, especially under 200 mM NaCl stress (Figure 7G). In transgenic plants and WT, salt stress induced MDA production with increasing NaCl concentration, but in transgenic plants, it was significantly lower than in WT (Figure 7H) in the same concentration of NaCl solution. Thus, the results showed that *KvCHX* enhanced salt tolerance in transgenic Arabidopsis seedlings.

### 3.7. KvCHX-Overexpressing Plants Grew Better Than WT under K^+^ Deficient Conditions

**Figure 8:**
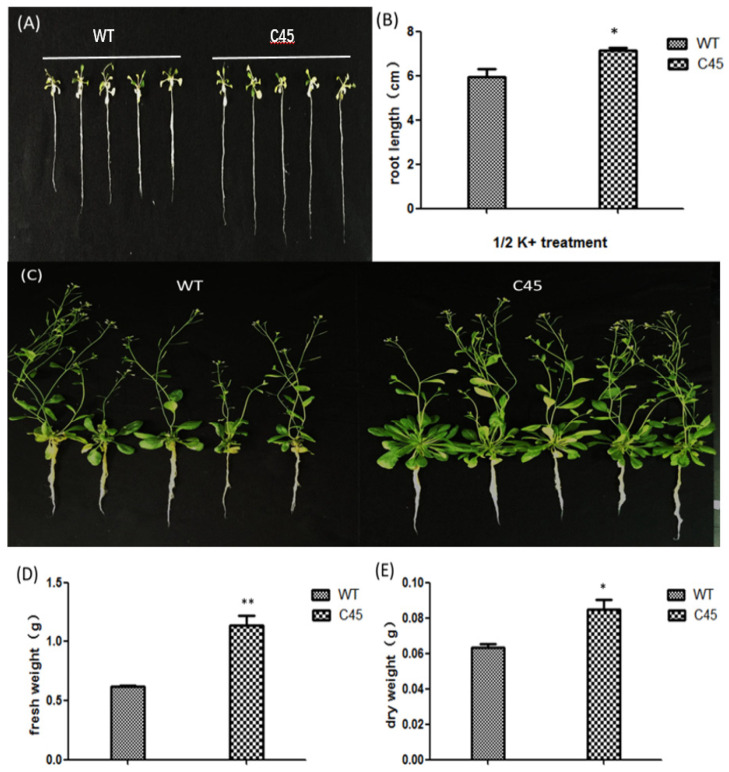
WT and transgenic Arabidopsis lines under deficient K^+^ treatment. (**A**) Seedlings of WT and transgenic (C45) plants were grown in basal nutrient solution for 10 days, and then transferred into 1/2 K^+^ nutrient solution for 20 days. (**B**) Root length of WT and transgenic plants. (**C**) Branch numbers of WT and transgenic plants. (**D**,**E**) Fresh weight and dry weight of transgenic lines and WT. (Values are mean ± SD for three independent replicates, n = 5.) *, *p* < 0.05; **, *p* < 0.01 in two-tailed Student’s *t*-tests.

After 20 days of culture in 1/2 K^+^ nutrient solution, WT and transgenic plants (C45) showed a different appearance. The transgenic plants were significantly vigorous, while WT shriveled under K^+^ deficient conditions (Figure 8). Moreover, the transgenic plants showed an increase in root length compared to the control plants (Figure 8A,B). The number of leaves and branches (Figure 8C), fresh weight (Figure 8D), and dry weight (Figure 8E) of the transgenic lines were higher than those of the WT lines. Transgenic plants grew better under K deficiency than WT.

### 3.8. Determination of Ion Contents of Transgenic Arabidopsis Lines under Deficient K^+^ Treatment and Treatment with NaCl

**Figure 9:**
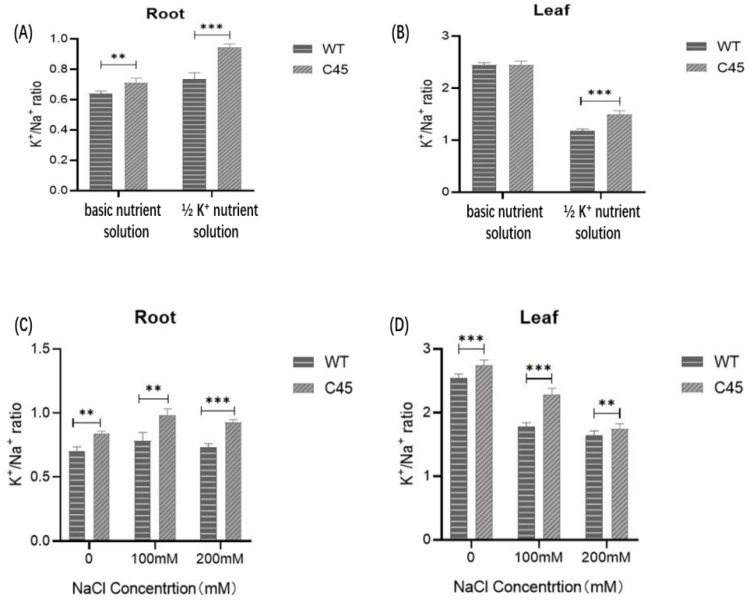
Comparison of the K^+^/Na^+^ ratios from roots and leaves between C45 transgenic plants and WT seedlings. (**A**) The K^+^/Na^+^ ratio from the roots of plants grown in basic nutrient solution and in 1/2 K^+^ nutrient solution. (**B**) The K^+^/Na^+^ ratio from the leaves of plants grown in basic nutrient solution and in 1/2 K+ nutrient solution. (**C**) The K^+^/Na^+^ ratio from the roots of plants under salt stress. (**D**) The K^+^/Na^+^ ratio from the leaves of plants under salt stress. Error bars indicate mean ± SD (values are mean ± SD for three independent replicates, *n* = 6). **, *p* < 0.01; ***, *p* < 0.001 in two-tailed Student’s *t*-tests.

The root and leaf K^+^/Na^+^ ratios of WT and C45 transgenic plants grown in basic nutrition solution were almost the same (Figure 9A). However, under ½ K^+^ treatment, the K^+^/Na^+^ ratio in the roots and leaves of C45 transgenic plants were both higher than that of WT (Figure 9B). In the roots, the K^+^/Na^+^ ratio of C45 transgenic plants was higher than that of WT by 28.69%, and that of C45 transgenic plants was higher than that of WT by 26.93% in the leaves. Similarly, the K^+^/Na^+^ ratio of C45 transgenic plants was higher in roots and leaves than that of WT. These results indicate that in the K^+^-deficient environment or salt stress conditions, the *KpCHX* gene could promote the absorption of K^+^ and the excretion of Na^+^ in transgenic Arabidopsis, which could help maintain a high level of intracellular K^+^/Na^+^ ratio and promote plant growth.

## 4. Discussion

Soil salinity is an important stress that limits plant growth and yield [33]. High concentrations of Na^+^ are toxic and have a negative effect on the growth of glycophyte plants. When plants grow in saline or alkaline soil environment, excessive sodium ions accumulate in the cytoplasm. A higher concentration of sodium ions is harmful for plant growth and disrupts enzymatic functions [34]. Halophytes have the ability to withstand salt stress and feature salt-tolerant genes to counter the adverse effects of salt stress [35,36]. In addition to the biosynthesis of osmoprotectants, activation of antioxidant enzymes, synthesis of polyamines and regulation of plant hormones, the survival strategies of halophytes also include other mechanisms under higher-saline soil [37].

*K. viginica* is a dicotyledonous halophyte that has been studied at the physiological level. However, the molecular mechanism of its tolerance to salt stress remains unknown. In this study, the *KvCHX* gene was isolated for the first time using the genome walking method. PCR and Western blot analysis showed that the *KvCHX* gene was integrated into the Arabidopsis genome, and the GFP-KvCHX fusion protein was expressed in transgenic plants. Via subcellular localization assay, KvCHX was localized on plasma membrane in the root. Transgenic *A. thaliana* showed better growth than WT under K^+^ deficiency treatment and salt stress.

In this study, *KvCHX* was specifically investigated to clarify its function of tolerance to salt stress. Phylogenetic and multiple comparison analyses revealed that *KvCHX* shared the highest homology with the *AtCHX17* gene, and coded for a putative cation/H^+^ transporter. Compared with WT, the overexpression of *KvCHX* promoted root development and increased the leaf number, fresh weight, and dry weight under salt tolerance, indicating that *KvCHX* enhanced the salt tolerance of transgenic Arabidopsis seedlings. With 1/2 K^+^ nutrient solution or NaCl stress, the K^+^/Na^+^ ratio of transgenic plants was higher than that of WT seedlings, and the K^+^/Na^+^ ratio of leaves was higher than that of roots. The results showed that *KvCHX* had a role in regulating the Na^+^ and K^+^ balance with 1/2 K^+^ nutrient solution and NaCl stress. The salt tolerance mechanism of the halophyte *K. virginica* may depend on the absorption and sequestration of Na^+^, K^+^ acquisition, and the maintenance of the K^+^/Na^+^ ratio to avoid the damage of NaCl toxicity.

Ion channels and transporters play an important role in plant salt tolerance. A number of ion transporters related functionally have been identified in plants via structural homology. Among them, members of the CPA gene superfamily are important transporters. They include the Na^+^/H^+^ exchanger (NHX), K^+^ efflux antiporter (KEA), and cation/H^+^ exchanger (CHX) families in plant genomes, with a conserved Na^+^/H^+^ exchanger domain [38,39,40]. The compartmentation of Na^+^ in the vacuoles by K^+^/Na^+^-specific NHX1-type antiporters and the maintenance of low Na^+^ in cytosol by HKT1-type transporters prevents or reduces the movement of Na^+^ to aboveground parts of plants [41]. Studies have shown that halophytes require high K^+^/Na^+^ ratios for normal cellular function [42].

The CPA2 family contains two subfamilies. A member of the CPA2 family, from Saccharomyces cerevisiae, appeared to mediate intracellular K^+^ flux. In fact, besides KHA from microorganisms, the *CHX* genes of Arabidopsis, soybean, rice, and other plants were identified as being involved in tolerance to salt stress, which has the function of cation/H^+^ transport [38]. *CHX* is a large family with 28 members in Arabidopsis [22], among which *AtCHX13* is considered to be a plasma membrane K^+^ transporter, playing a role in increasing plant K^+^ uptake in a potassium-deficient environment [43]. *AtCHX16-20* have different regulatory effects on K^+^ and pH homeostasis in different cellular compartments [44]. A variety of environmental stresses, including high salt, potassium deficiency, ABA, and acidic mediators, can up-regulate *AtCHX17* transcripts [24]. The *AtCHX17* knockout mutant lines accumulate less K^+^ in response to salt stress and K^+^ starvation than the wild type. CHX20 is mainly localized to the membrane of the endosomal system and not only maintains K^+^ homeostasis, but also affects the pH under certain conditions [14]. *AtCHX21* and *AtCHX23* are involved in K^+^ homeostasis in the female gametophyte, and *AtCHX21* is also involved in regulating xylem Na^+^ concentration and Na^+^ accumulation in leaves [45]. According to previous research on the model plant *Arabidopsis*, *AtCHX17-19*, which is highly homologous to the *KvCHX* gene, is located on the membrane system, which is highly similar to the localization of *KvCHX* in our study [14]. *GmSALT3* belongs to the *CHX* family and is predominately expressed in root phloem- and xylem-associated cells under both saline and non-saline conditions [46]. *GmSALT3* improves the ability of salt-tolerant near-isogenic soybean lines under saline stress through preventing excessive ROS accumulation in roots, and potentially modulating Ca^2+^ signaling, vesicle trafficking, and the formation of diffusion barriers [15]. GmSALT3 confers net shoot exclusion for both Na^+^ and Cl^−^ and improves salt tolerance in soybeans. *GmCHX1* from salt-tolerant soybeans was shown to protect plants via Na^+^ exclusion under salt stress [47]. *GmCHX20a* and *GmCHX1* might work complementally through a concerted effort to address both osmotic stress and ionic stress as a result of elevated salinity. An overexpression of *GsCHX19.3* in Arabidopsis improved plant tolerance to under saline–alkali stress by reducing the Na^+^ concentration and increasing the K^+^/Na^+^ ratio [48]. *OsCHX14* is regulated by the JA signaling pathway, capable of transporting K^+^, Rb^+^, and Cs^+^ in vivo, and plays an important role in K^+^ homeostasis during flowering in rice [49].

Halophytes prevent Na^+^ accumulation in the cytoplasm through a variety of mechanisms, including Na^+^ extrusion and/or the intracellular compartmentalization of Na^+^, along with the recirculation of Na^+^ out of the shoot and the up-regulation of Na^+^/H^+^ antiporters in the plasma membrane [50,51]. Here, we demonstrate that the *KvCHX* gene participates in regulating K^+^/Na^+^ to reduce damage to *K. virginica* seedlings under salt stress. Our study shows that the *KvCHX* gene confers salt tolerance to *A. Thaliana* by taking up and translocating K^+^ to withstand salt stress. The *KvCHX* gene may play a key role in selective K^+^ accumulation and transport from roots to leaves at the cellular and whole-plant levels. These results provide genetic and biochemical evidence that the *KvCHX* protein plays a major role in the balance between K^+^ influxes and possibly Na^+^ modulation via regulating different transporters, directly or indirectly.

Improving salt tolerance is an important goal in plant breeding. Genes associated with halophyte survival strategies can be used to modify glycophytes through protecting and maintaining the function and structure of cellular components. In the current research, we report that the *CHX* gene from *K. virgineca*, a salt marsh halophyte, maintains Na^+^ and K^+^ homeostasis in transgenic Arabidopsis lines. *KvCHX* encodes a K^+^, Na^+^/H^+^ exchanger, which not only mediates K^+^ uptake, but also has a role in regulating cellular ion homeostasis through the expulsion of Na^+^ as cation antiporters under NaCl stress. At present, GmSALT3, as a *CHX* family gene in soybeans, has been shown for the first time to improve plant salt tolerance by promoting the recycling of Cl^−^ in the phloem. Although our study reveals the potential mechanism by which the *KvCHX* gene enhances the salt stress ability of Arabidopsis, further research is still needed [14]. It is not clear which initial signals can directly initiate the upstream and downstream cascade involving K^+^ activation and Na^+^ expulsion, but it is conceivable that *KvCHX* or other related cation exchangers may have significant effects on the osmotic pressure, volume, and pH of plant cell compartments. In addition, *CHX* may also be involved in the polarized growth of plants, which is of great significance to follow-up research. Therefore, future work needs to focus on the exploration of molecular signals to reveal new insights into K^+^/Na^+^ regulation for plants in response to abiotic stress.

## Figures and Tables

**Table 1:** Primers used for the genome walking and sequencing of the *KvCHX* gene.

GSP1-5′-2	5′-CCCAAAACTTCAGGACCAGG-3′	GSP1-3′-2	5′-CCTGGTCCTGAAGTTTTGGG-3′
GSP1-5′-3	5′-GCTTCGTGATTGTCGTGTCC-3′	GSP1-3′-3	5′-CAATCTTTTCGTGGTGGGCC-3′
GSP2-5′-1	5′-GGGGAGAAGAGTTTGTTCCTG-3′	GSP3-5′-1	5′-CAACTATCTCGCCGATGACC-3′
GSP2-5′-2	5′-TACGGGCTAACACAGGGAAG-3′	GSP3-5′-2	5′-GCGAGGATACGAGTGAGTGC-3′
GSP2-5′-3	5′-AAGGTGTCCAGCACCGTTAG-3′	GSP3-5′-3	5′-GTACCGCTATCACCGACGAA-3′
RV-M	5′-GAGCGGATAACAATTTCACACAGG-3′	M13-47	5′-CGCCAGGGTTTTCCCAGTCACGAC-3′

Note: The first genome walking primers of 5′-end flanking sequence: GSP1-5′-1~GSP1-5′-3. The second genome walking primers of 5′-end flanking sequence: GSP2-5′-1~GSP2-5′-3. The third genome walking primers of 5′-end flanking sequence: GSP3-5′-1~GSP3-5′-3. The genome walking primers of 3′ -end flanking sequence: GSP1-3′-1~GSP1-3′-3. The primers for the sequencing of genome walking products: RV-M and M13-47.

**Table 2:** The PCR programs used in TAIL-PCR reactions.

PCR Reaction	Cycle No.	Thermal Condition
The first round of PCR(GSP-5’-1/AP)	1	94 °C 1min, 98 °C 1min;
	5	94 °C (30 s), 60 °C (1 min), 72 °C (3 min)
	1	94 °C (30 s), 25 °C (3 min), 72 °C (3 min)
	15	94 °C (30 s), 60 °C (1 min), 72 °C (3 min); 94 °C (30 s), 60 °C (1 min), 72 °C (3 min); 94 °C(30 s), 44 °C (1 min), 72 °C (3 min)
	1	72 °C (10 min)
The second round of PCR(GSP-5′-2/AP)	15	94 °C (30 s), 60 °C (1 min), 72 °C (3 min); 94 °C (30 s), 60 °C (1 min), 72 °C (3 min); 94 °C (30 s), 44 °C (1 min), 72 °C (3 min)
	1	72 °C (10min)
The third round of PCR(GSP-5′-3/AP)	15	94 °C (30 s), 60 °C (1 min), 72 °C (3 min); 94 °C (30 s), 60 °C (1 min), 72 °C (3 min); 94 °C (30 s), 44 °C (1 min),72 °C (3 min)
	1	72 °C, 10 min

## Data Availability

Data are contained within the article.
